# Paper-Based Lateral Flow Device for the Sustainable Measurement of Human Plasma Fibrinogen in Low-Resource Settings

**DOI:** 10.1021/acs.analchem.1c03665

**Published:** 2021-10-07

**Authors:** Jerro Saidykhan, Laura Selevic, Stefano Cinti, Jennifer E. May, Anthony J. Killard

**Affiliations:** † Centre for Research in Biosciences (CRIB), Department of Applied Sciences, University of the West of England, Coldhar-bour Lane, Bristol BS16 1QY, U.K.; ‡ Department of Pharmacy, University of Naples “Federico II”, Via Domenico Montesano 49, Napoli 80131, Italy

## Abstract

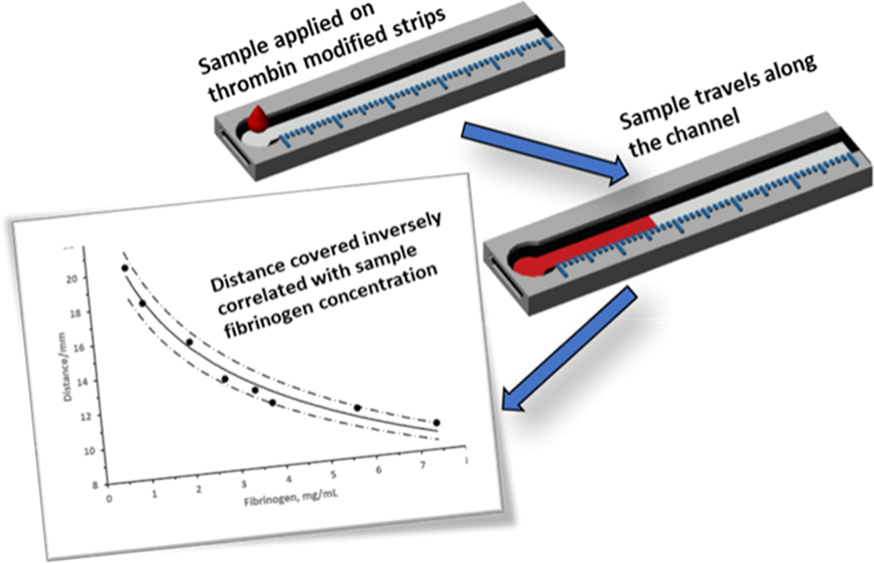

Fibrinogen concentration is a major determinant of both clotting and bleeding risk. Clotting and bleeding disorders cause extensive morbidity and mortality, particularly in resource-poor and emergency settings. This is exacerbated by a lack of timely intervention informed by measurement of fibrinogen levels under conditions such as thrombosis or postpartum haemorrhage. There is an absence of simple, rapid, low-cost, and sustainable diagnostic devices for fibrinogen measurement that can be deployed in such environments. Paper-based analytical devices are of significant interest due to their potential for low-cost production, ease of use, and environmental sustainability. In this work, a device for measuring blood plasma fibrinogen using chromatography paper was developed. Wax printing was used to create hydrophobic structures to define the test channel and sample application zone. Test strips were modified with bovine thrombin. Plasma samples (22 μL) were applied, and the flow rate was monitored over 5 min. As the sample traversed the strip, clotting was induced by the conversion of soluble fibrinogen to insoluble fibrin. The flow rate and distance travelled by the sample were dependent on fibrinogen concentration. The device was able to measure fibrinogen concentration in the range of 0.5–7.0 ± 0.3 mg/mL (*p* < 0.05, *n* = 24) and had excellent correlation with laboratory coagulometry in artificial samples (*r*^2^ = 0.9582, *n* = 60). Devices were also stable at 4–6 °C for up to 3 weeks.

Fibrinogen is a 340 kDa glycoprotein that circulates in blood in a normal range of 2–4 mg/mL. Its conversion to fibrin is the basis of clot formation during coagulation.^1^ Fibrinogen is also involved in primary haemostasis where it facilitates platelet adhesion and aggregation at the wound site.^2^ Low fibrinogen levels may result in postpartum haemorrhage,^3^ pregnancy loss, and intracranial and joint bleeding, while elevated fibrinogen may be a risk factor in thrombophilia.^4^ Fibrinogen is an important biomarker for assessing haemostatic function and diagnosing haemostatic disorders. Bleeding and clotting disorders cause extensive morbidity and mortality, especially in resource-poor settings, which may lack access to suitable testing methods. There are several routine hospital-based methods for fibrinogen in whole blood and plasma including the Clauss assay,^5^ viscoelastic assays,^6,7^ dry haematology methods,^8^ and prothrombin time-derived fibrinogen assay.^5^ However, such approaches are not suitable for point-of-care use in resource-limited environments. A rapid, inexpensive, and easy-to-use fibrinogen assay would significantly enhance the diagnosis and treatment of coagulation disorders.

Lateral flow assays (LFAs) have been widely used in point-of-care diagnostic applications.^9^ The combination of lateral flow to drive assay processes with visual reading of the test result produces low cost, easy-to-use tests with no instrumentation. LFA devices based on polymer microfabrication have been developed for the measurement of fibrinogen.^4^ While devices such as these may be effective for instrument-free point-of-care diagnostics, they still lack environmental sustainability and may result in the accumulation of plastics in the environment. Paper-based LFAs based predominantly on nitrocellulose substrates are used widely for many rapid assays for pregnancy^10^ and COVID-19.^11^ Porous paper, for example, chromatographic paper, has re-emerged as an attractive substrate in bioanalytical devices because of its many positive features including very low cost, abundant availability in many forms, biocompatibility, biodegradability, disposability, ease of modification, and controlled porosity for driving fluid flow.^12,13^ Although in use since the 1950s for the determination of glucose levels in urine,^12^ there has been a significant resurgence in cellulose paper-based technologies and devices in recent years due to these benefits, and such devices are again being widely studied in many applications. The application of paper-based devices in clinical diagnostics, including blood coagulation testing, has been recently reviewed.^14^

Paper-based devices have recently been applied to fibrinogen measurement. An initial approach employed a vertically suspended paper substrate, with fibrinogen levels determined from the distance travelled by the sample up the strip.^15^ However, the orientation of the device and the need for a fluid reservoir make it impractical for point-of-care use. Another approach utilized the permeability of the paper to fibrinogen. Increased fibrinogen concentration led to a decrease in permeability and therefore reduced the distance travelled by the sample. This method could measure fibrinogen in both plasma and whole blood and had an assay time of 4 min but was only suitable for samples with low fibrinogen concentrations (<1.6 mg/mL).^16^

A similar approach exploited the resulting differences in hydrophobicity of the paper when exposed to samples of different fibrinogen concentrations, which altered the distance travelled by a dye applied to the paper following sample application.^17^ This method could only measure fibrinogen at <2 mg/mL. Both methods require multistep procedures and have the potential to be prone to many interferences such as variations in sample viscosity. Dielectrophoresis has been recently used in conjunction with a paper-based assay for measuring fibrinogen.^18^ The device had a dynamic range from 1.27 to 5.08 mg/mL, high precision, and good agreement with well-established routine laboratory methods. However, it required complex instrumentation and fabrication methodologies, making it unsuitable for use in low-resource environments. There is still a significant need for simple, robust, and sustainable devices for measuring fibrinogen in low-resource and emergency settings.

In this work, a paper-based lateral flow device was developed for the measurement of fibrinogen. The device employed wax-printed paper-based strips to define microfluidic channels, with thrombin deposited as a coagulation reagent. The device was rigorously optimized for measurement across a wide dynamic assay range and validated using coagulometry.

## Materials and Methods

### Materials

Whatman no. 1 chromatography paper (460 × 570 mm, 1001-917), Chromozym TH, and lyophilized fibrinogen from human plasma (F3879) were from Sigma-Aldrich, UK. Lyophilized bovine thrombin (50 NIH units/mL) was from Clauss Fibrinogen 50 kit (5556), and calibration plasma containing approx. 2.8 mg/mL fibrinogen (5185) was from Helena Biosciences, UK. Defibrinated plasma (approx. 1.5 mg/mL fibrinogen) was purchased from BioIVT, UK, while HemosiL low-fibrinogen control plasma with approx. 0.75 mg/mL fibrinogen (0020004200) was from Instrumentation Laboratory, UK. Yumizen G FIB 2 (130036383) and Yumizen G imidazol (130036385) reagents were from Horiba, UK. Aluminum foil Mylar bags (X000Y9UA0L, 12 × 8 cm) were from Fresherpack, UK, while desiccant silica gel sachets (10 g) were from CelloExpress.

### Device Fabrication

**Figure 1:**
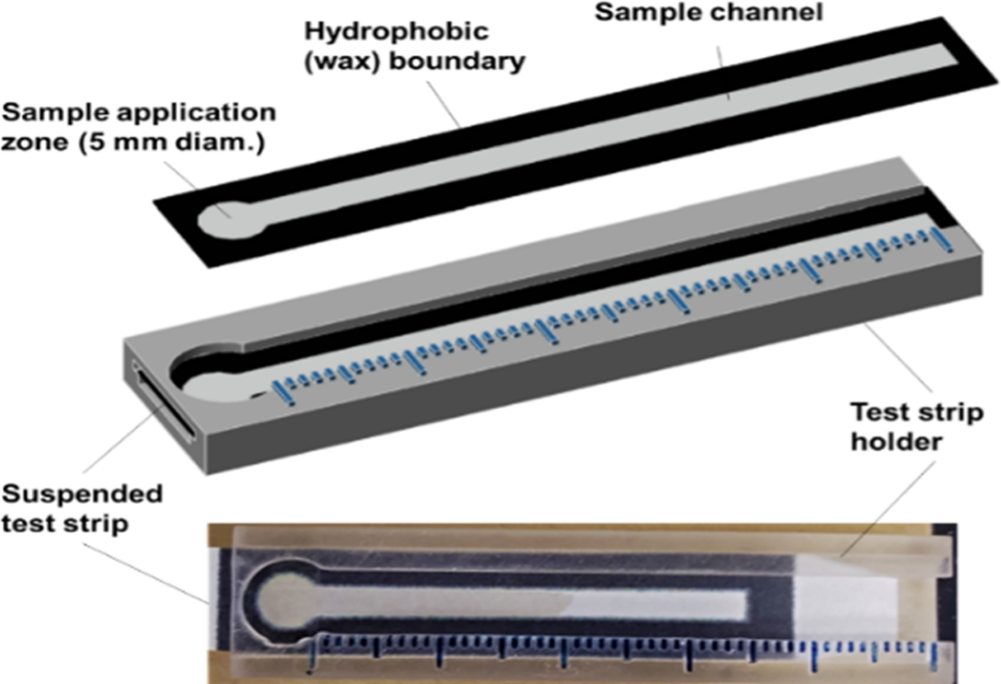
Paper-based lateral flow device for measuring fibrinogen in plasma. (Top) Paper strip design layout; (Middle) design layout of strip in the holder; and (Bottom) photograph of the strip suspended in the holder.

Whatman no. 1 chromatography paper was cut into A4 sheets. Lateral flow strip configurations were designed using Microsoft PowerPoint 2016 and printed using a ColorQube 8570 wax printer (Xerox Corporation, Malaysia). Strip designs were typical of that shown in Figure 1, with a circular sample application zone and a linear test channel. Two designs were employed in this work: 5 × 80 mm and 3 × 36 mm. The printed strips were heated at 100 °C for 2 min to melt the wax and form a hydrophobic seal^19^ and were then cut from the sheet.

Paper-based strips were suspended on a wire rack for application of thrombin reagent. Lyophilized bovine thrombin was reconstituted and diluted with deionized water and activity was measured using a chromogenic assay.^20^ Thrombin in the range of 10–50 NIH units/mL was deposited onto strips at the sample application zone and typically allowed drying for approx. 40 min at room temperature. A final thrombin concentration of 30 NIH units/mL was used for assay validation.

### Preparation of Fibrinogen Standards and Artificial Samples

Assay development and optimization was typically performed using human plasma with a normal concentration of fibrinogen (approx. 2.8 mg/mL). For calibration, fibrinogen standards in the range of 0.8–7.4 mg/mL were prepared by reconstituting a low fibrin content plasma (0.8 mg/mL) and dissolving lyophilized fibrinogen in plasma with 1.5 mg/mL. For device validation, artificial plasma samples were constructed from commercial materials. Twenty artificial plasma samples of unknown concentration were prepared in the low (eight samples of approx. 1 mg/mL), normal (eight samples of approx. 3 mg/mL), and high fibrinogen concentration (four samples of approx. 5 mg/mL) ranges.

### Determination of Fibrinogen Concentration

The concentration of fibrinogen in the calibration standards and artificial samples was determined using the Yumizen G200 coagulation analyzer (Horiba, UK). Samples were diluted 1 in 5 (≤1.0 g/L fibrinogen), 1 in 10 (1 to 5 g/L fibrinogen), or 1 in 20 (≥5 g/L fibrinogen) in imidazole buffer, according to the manufacturer’s instructions.

### Storage of Strips

For storage and stability studies, the assay strips were placed in aluminum/Mylar foil bags with 10 g of silica gel desiccant sachets and sealed under partial vacuum.

### Flow Rate Measurements

For testing, strips were placed inside an ad hoc 3D-printed holder in which the wax edges of the strips were placed on elevated rails, allowing the paper strip to be freely suspended (Figure 1). The 3D holder had an opening in the top to allow sample deposition and visual monitoring of fluid flow. The holder was also equipped with a millimetric scale to measure the distance travelled by the sample. All flow rate measurements were performed at 37 °C on a flat heating block on which the strip holder was placed. Sample reagents were also pre-equilibrated for 5 min before use. Samples were applied to the sample application zone, and flow rates were captured using a video recorder, typically for 5 min.

## Results and Discussion

The main types of paper used as substrates in analytical devices are cellulose-based chromatography paper and nitrocellulose.^21^ Chromatography paper is a low-cost, biodegradable cellulosic material and was selected as preferable for use in this work due to these properties. With regard to deposition of assay reagents onto paper substrates, several methods have been used, such as drop-casting, inkjet printing, and spray-coating.^13^ While automated techniques such as inkjet printing and spray-coating allow a great deal of control and precision for scale-up and mass production, they add significant complexity to the development process, while drop-casting is simple and highly effective and was effectively employed throughout this work.

### Effect of the Sample and Reagent Composition on the Sample Flow Rate

To develop a blood coagulation assay device based on lateral flow principles, it is essential to account for all factors that might influence the fluid dynamics of the sample. In the case of this device, the most significant influences on the sample flow rate are likely to be the impact of coagulation itself, the viscosity of the sample, and the hydrophobicity, wettability and solubility properties of the substrate and any deposited reagents as they come into contact with the advancing sample.

To determine whether any effects on flow rate were as a result of coagulation, viscosity, or other factors, a number of clotting and nonclotting controls were performed to quantify these. With respect to sample viscosity and appropriate controls, human plasma viscosity is typically in the range of 1.1–1.3 mPa s at 37 °C,^22^ while serum is 1.4 to 1.8 mPa^23^ and so have the potential to act as positive (clotting) and negative (nonclotting) controls, respectively. However, care should be taken with serum as a negative control due to the potential presence of residual fibrinogen and activated coagulation factors.^24^ Preliminary studies had investigated the appropriate volumes of assay reagents and test samples to use in conjunction with the paper-based assay strips, which would sufficiently wet but not oversaturate the entire strip. These were 90 and 70 μL, respectively, for channels of 5 × 80 mm.

**Figure 2:**
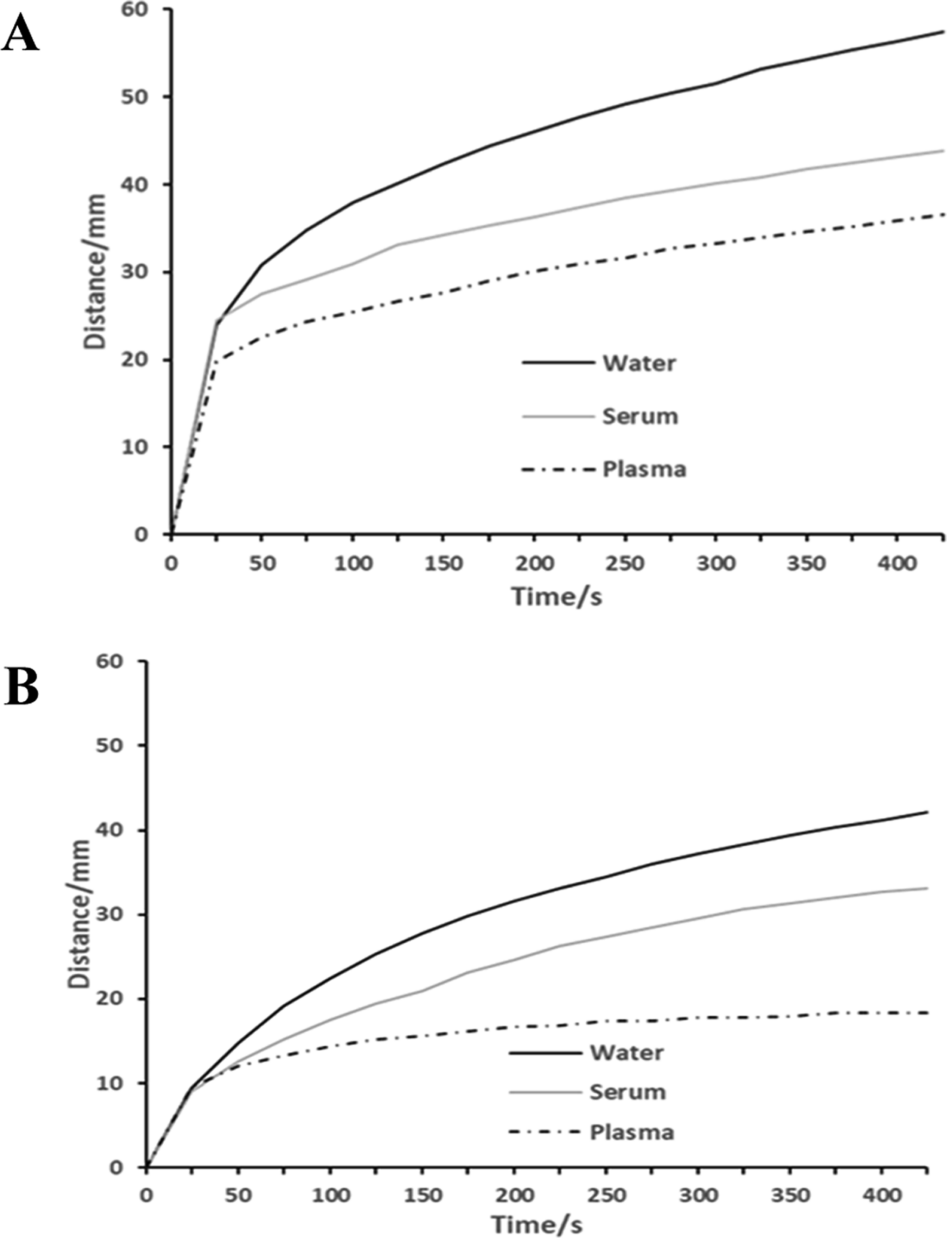
Flow rates of sample viscosity and clotting controls (70 μL) on (A) buffer-modified and (B) thrombin-modified strips. Paper assay strips (5 × 80 mm) were modified with 90 μL of (A) Owren’s buffer or (B) thrombin (50 NIH/mL).

Strips were modified with 90 μL of either Owren’s buffer or thrombin at an initial concentration of 50 NIH/mL. Sample viscosity and clotting controls of water, human plasma, and serum (70 μL) were applied to modified, dried strips, and the flow rates were measured (Figure 2). The flow rate of all samples was reduced on the thrombin-modified strips (Figure 2B) in comparison with buffer-modified strips (Figure 2A), demonstrating the impact of hydrophobicity, wetting, and solubility effects on flow dynamics. The flow rate of water was the greatest on both surfaces, due to its lower viscosity. While plasma flow rates were lower than serum on both surfaces, this difference was significantly greater on the thrombin-modified strips, which could be attributed to clotting and which suggested a dynamic assay measurement range under these assay conditions of approx. 12 mm between clotting and nonclotting controls.

### Assay Optimization of Thrombin Concentration and Sample Volume

**Figure 3:**
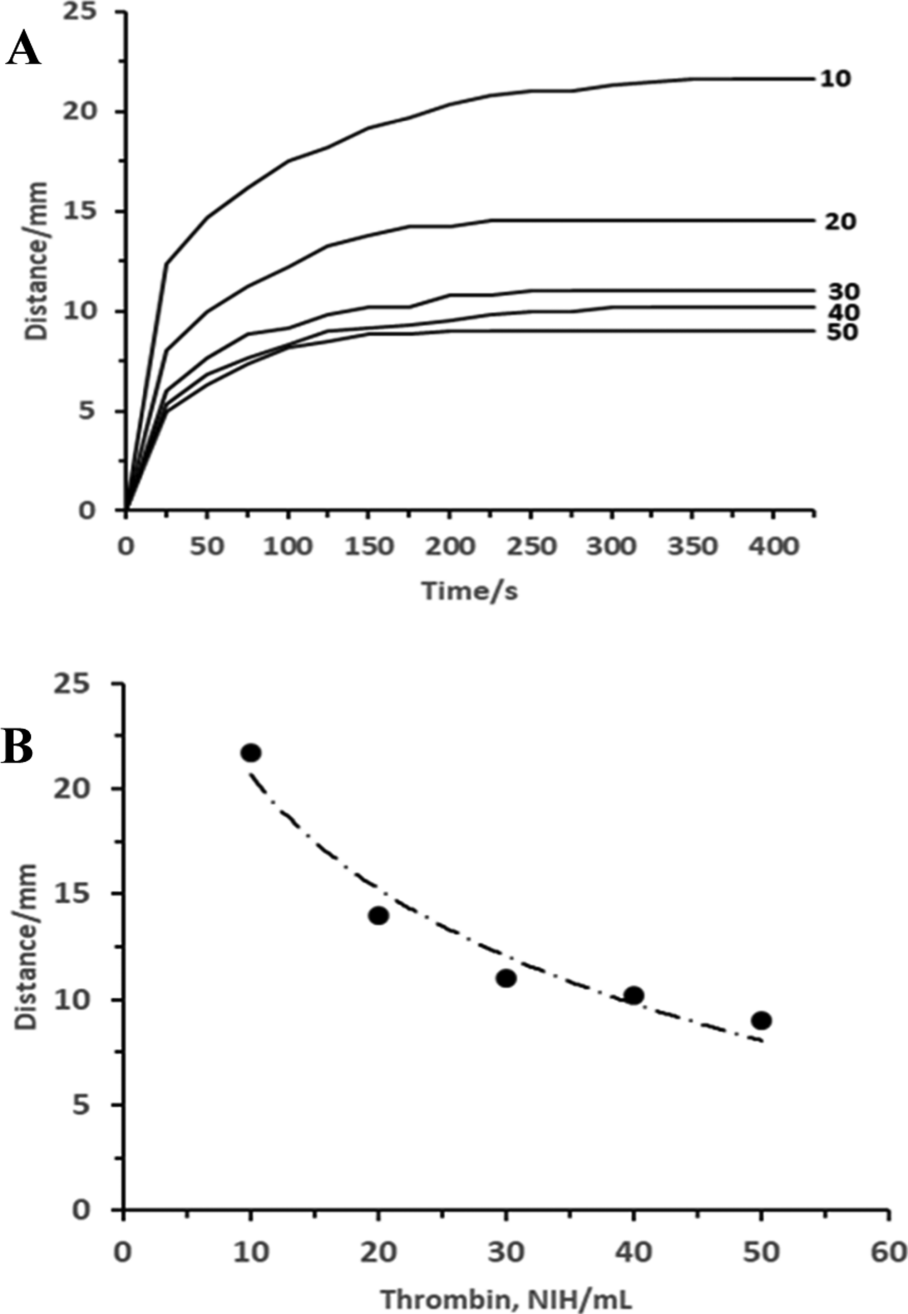
(A) Flow rates of human plasma (20 μL) on strips (3 × 36 mm) modified with 27 μL of thrombin at 10–50 NIH/mL. (B) Effect of thrombin activity on the distance travelled by plasma samples at 37 °C.

The assay was further miniaturized to strip dimensions of 3 × 36 mm and sample and reagent volumes of 20 and 27 μL, respectively, reducing material and reagent usage. Following the initial investigation of thrombin at 50 NIH/mL, further analysis was performed to determine the effect of thrombin concentration on assay performance. The flow rates of human plasma were analyzed on strips modified with thrombin at 10 to 50 NIH/mL (Figure 3A). The plasma flow rate and the distance travelled by the sample after 5 min were inversely proportional to thrombin concentration (Figure 3B), demonstrating more rapid fibrin clot formation and arrest of flow in the presence of higher thrombin concentrations.

While the flow rate and distance travelled were the lowest for 50 NIH/mL, the overall response to thrombin concentration was curvilinear, with only fractional decreases between 30 and 40 NIH/mL, suggesting that at 50 NIH/mL, the effect on clotting was close to maximum. However, while 50 NIH/mL might be close to optimal for assay speed, it might not be so for other assay parameters such as range, sensitivity, linearity, and precision. Normal human plasma, such as the one used here, has a fibrinogen concentration of approx. 2.8 mg/mL.^25^ However, under hypo- and hyperfibrinogenemic conditions, this can vary from <1 to >5 mg/mL.^26,27^ It is critical that optimization studies ensure that the assay can operate across this range. Optimization of thrombin concentration solely at 2.8 mg/mL fibrinogen might limit assay range, particularly at lower fibrinogen concentrations.

The effect of thrombin activity on assay performance was evaluated at fibrinogen concentrations of 1.47 (low), 3.70 (normal), and 6.36 (high) mg/mL and their analytical performance parameters are summarized in Table 1. The assay at 30 NIH/mL had the highest sensitivity and best precision, while also having good linearity, and so was adopted for further assay development. Higher activity of thrombin leads to faster clotting rates but may have the overall effect of narrowing the dynamic range and reducing assay sensitivity. Lower concentration of thrombin, on the other hand, may improve sensitivity but with reduced precision. Thrombin at 30 NIH/mL appeared to be in sufficient excess over fibrinogen across the full assay range.

**Table 1:** Analytical Performance Parameters for Fibrinogen Calibration Assays Across a Range of Thrombin Concentrations

thrombin (NIH/mL)	slope (mg/mL/mm)	*r*^2^	CV (%)
20	–4.379	0.842	10.29
30	–6.449	0.999	5.88
40	–5.427	0.947	13.24
50	–4.059	0.985	16.52

### Assays Based on 20 μL Fibrinogen Standards of 1.47, 3.70, and 6.36 mg/mL

Another parameter critical to analytical performance is sample volume. Minimizing sample volume is a key parameter to reduce the impact of testing on patients. However, reducing sample volume can lead to increased errors in the volume of the sample delivered to point-of-care devices.^28^ Analytical methods require precisely optimized sample volumes, which were investigated here. For this assay, in particular, reduced sample volume is likely to lead to reduced linearity and sensitivity, while increased sample volumes may encourage bulk flow along the strip, rather than capillary action, which may reduce precision. From preliminary studies, the optimum sample volume seemed to lie between 20 and 30 μL. Sample volumes of 20, 22, 24, and 26 μL were investigated at three fibrinogen concentrations and their analytical parameters are summarized in Table 2. The sensitivity indicated by the slope, the linearity indicated by the correlation coefficient (*r*^2^), and the precision indicated by % CV were considered indicators of performance and hence used here in the optimization of sample volume, reagent activity, and reagent volume. A 26 μL sample volume achieved good sensitivity (slope = −6.561) but poor linearity (*r*^2^ = 0.680) and precision (CV = 13.49%). A sample volume of 24 μL gave the best linearity (*r*^2^ = 0.999), although poor precision (CV = 11.57%). With a slope of −5.373, an *r*^2^ of 0.954, and a CV of 4.5%, a 22 μL sample volume appeared to have the best combination of good sensitivity, linearity, and precision and was therefore used for final assay demonstration. The performance parameters of 20 μL sample volume were all below those of 22 μL.

**Table 2:** Analytical Performance Parameters for Fibrinogen Calibration Assays Across a Range of Sample Volumes

sample volume/μL	slope (mg/mL/mm)	*r*^2^	CV (%)
20	–4.701	0.927	6.51
22	–5.373	0.954	4.50
24	–4.579	0.999	11.57
26	–6.561	0.680	13.49

### Assays Based on 30 NIH/mL Thrombin and Fibrinogen Standards of 1.10, 2.84, and 5.68 mg/mL

#### Final Assay Calibration

**Figure 4:**
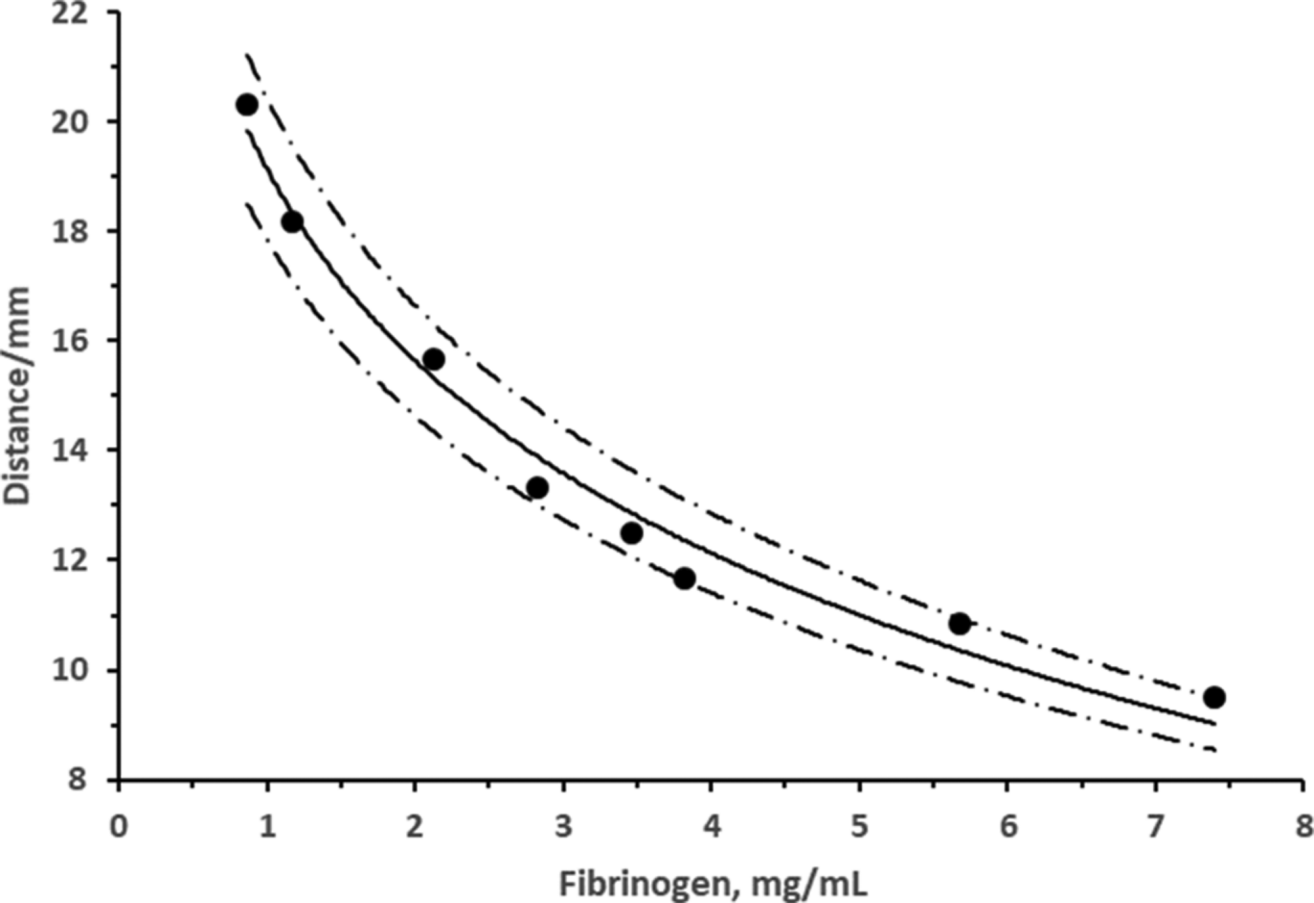
Calibration curve of concentration of fibrinogen standards as determined by Yumizen G200 versus distance travelled after 300 s for the optimized paper-based fibrinogen assay. Assay based on deposition of 27 μL of 30 NIH/mL thrombin and 22 μL of the sample (*n* = 3 repeats). Equation of the line, *y* = −5.058 ln(*x*) + 19.143 (*r*^2^ = 0.9822). Dashed lines: 95% confidence.

Fibrinogen standards (22 μL) with concentrations in the range 0.87–7.4 mg/mL (as determined by Yumizen G200) were tested on paper-based strips modified with 30 NIH/mL thrombin (27 μL) (*n* = 3) (Figure 4). The distance travelled at 300 s was from 20.33 mm at 0.87 mg/mL to 9.50 mm at 7.4 mg/mL. The response was curvilinear, with excellent fit to a semilogarithmic plot with a slope of −5.058 and an *r*^2^ of 0.9822. Min., max., and mean coefficients of variation (*n* = 3) were 1.84, 9.67, and 5.19%, respectively. At 95% confidence, a measurement of 2.8 mg/mL would be within the range of 2.5–3.2 mg/mL.

#### Assay Validation

Validation of the assay was performed during the global COVID-19 pandemic. As a result, there was no access to clinical samples for validation studies. To overcome this, artificial plasma samples were constructed from combinations of commercial materials from various sources.

To validate the device for measuring fibrinogen in human plasma, 20 plasma samples were prepared using mixtures of low- and normal-fibrinogen plasmas, as well as plasmas supplemented with lyophilized fibrinogen to create unknowns with approximate fibrinogen concentrations in the region of 1, 3, and 5 mg/mL.

**Figure 5:**
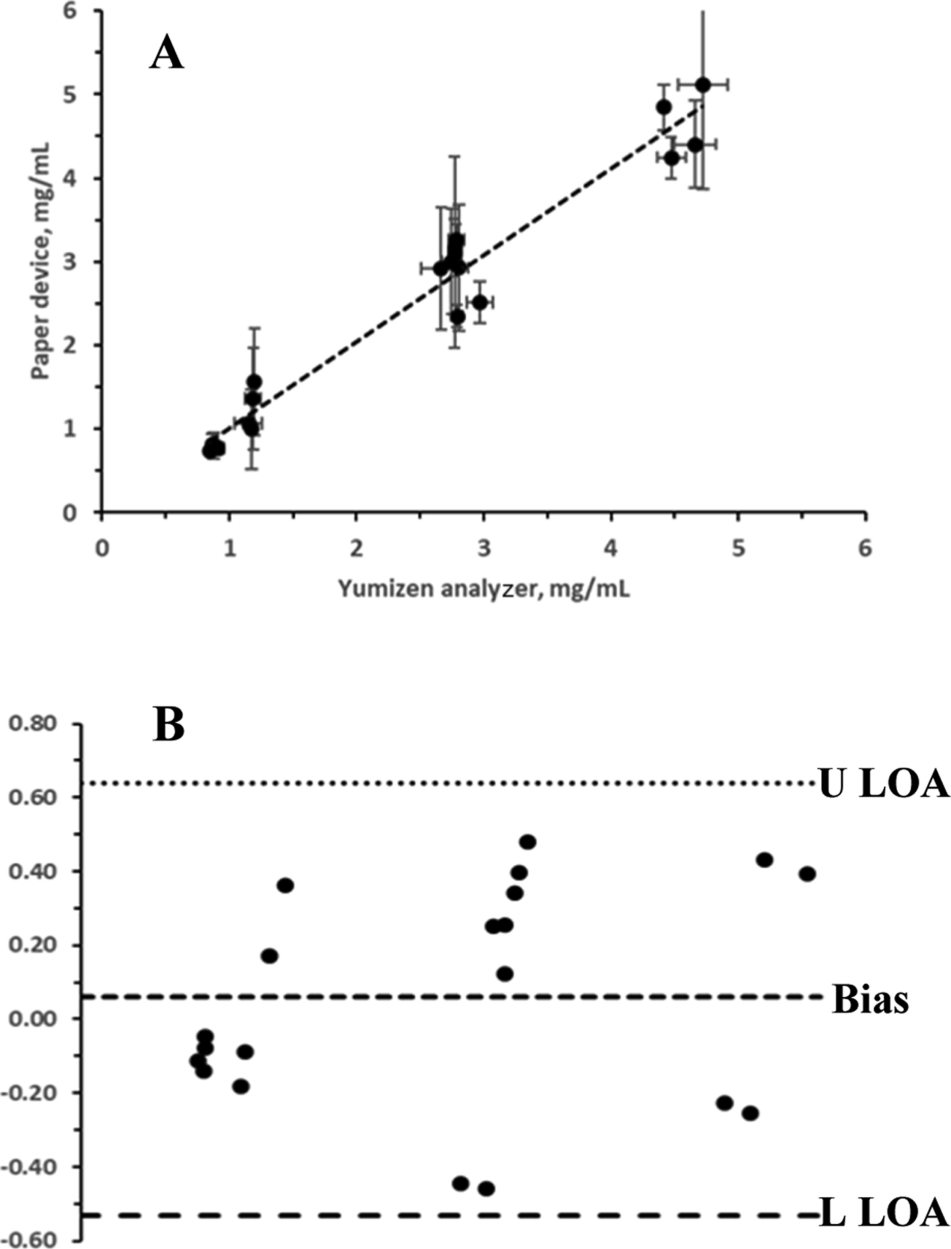
Validation of the paper-based fibrinogen assay device in artificial plasma samples. (A) Correlation between the paper-based device and Yumizen G200 fibrinogen assay reference method. A slope of 1.03, an intercept of 0.02, and an *r*^2^ of 0.9582 (20 samples, 3 repeats). (B) Bland–Altman shows that all of the data points fall within the range between the upper limit of agreement (U LOA) and the lower limit of agreement (L LOA), signifying adequate agreement between the two methods.

Each sample was tested simultaneously with the paper-based device (*n* = 3 repeats), applying the calibration curve established in Figure 4, and with the Yumizen coagulation analyzer G200. The results from the two methods were compared using linear least-squares regression (Figure 5A) and Bland–Altman analysis (Figure 5B). There was excellent agreement between the developed and reference method, with a slope of 1.03, a very small offset of 0.02 mg/mL, and good linearity across the analytical range from approx. 1 to 5 mg/mL with a correlation coefficient of 0.9582.

With a wide dynamic range of 0.5–7 mg/mL, coupled with adequate agreement with a well-established routine reference method, the performance of this device is excellent compared to similar methods recently published. It was capable of reliable measurement of fibrinogen at all concentrations across the full range found in human plasma and is suitable for both hyper- and hypofibrinogenemic conditions, which is in contrast to the very narrow assay range of other recent work.^16,17^ In addition, the method used here was a simple, robust, and single step, making it more user-friendly, while its design makes it less-prone to errors and interference. It is also compact, portable, and instrument-free, making it highly suited to point-of-care application.

#### Storage Stability and Operating Temperature

A major challenge for diagnostic devices is their stability in a range of operational conditions. Packaging and storage can add cost and complexity to device production and usage, and devices need to withstand variations in temperature and humidity over prolonged periods of time. To assess device stability, assay strips were prepared, placed in sealed aluminum foil pouches with desiccant, and stored for up to 28 days in a range of storage temperatures. Strips were then tested for their flow rates and clotting distances using normal plasma (Table 3).

**Table 3:** Effect of Storage Conditions on Clotting Properties of Paper-Based Fibrinogen Assay Strips

storage conditions	distance travelled by normal plasma/mm after number of days storage (*n* = 3)				
	day 0	day 7	day 14	day 21	day 28
freezer (−15 to −18 °C)	13.17 ± 0.76	12.83 ± 0.76	13.00 ± 1.00	12.67 ± 0.58	14.67 ± 3.40
fridge (4 to 6 °C)		13.50 ± 1.50	12.50 ± 0.50	13.00 ± 0.00	15.00 ± 3.12
ambient (18 to 23 °C)		13.00 ± 0.00	11.83 ± 1.76	11.17 ± 0.76	11.67 ± 1.04
oven (37 °C)		10.83 ± 0.58	12.67 ± 0.29	12.33 ± 1.04	10.67 ± 0.76

The distance travelled by plasma on strips stored in the fridge and freezer appeared to be within the acceptable range for 3 weeks. However, at the end of the fourth week, measurements from these strips started to fall outside the reference range and also had significantly increased variability. Strips stored at ambient temperature gave acceptable measurements after the first week (13 mm), after which values were shorter than the reference range, which may be due to increased paper and reagent hydrophobicity due to increased desiccation. Strips stored at 37 °C were also below the reference range in the first, third, and fourth weeks and may be due to changes in hydrophobicity and thrombin activity. However, without any further stabilization, the paper devices appeared stable for up to 3 weeks at 4 to 6 °C. This demonstrates the potential for long-term stabilization of thrombin activity on paper to be achievable with the addition of stabilizers, excipients, and processing techniques such as lyophilization.

**Figure 6:**
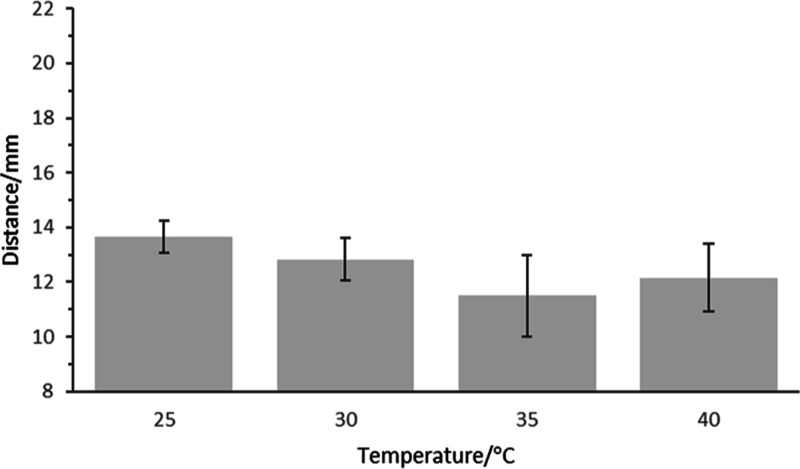
Effect of operating temperature. Optimized assay strips were operated from 25 to 40 °C in normal plasma (*n* = 3).

To be viable as an instrument-free device, particularly in low-resource settings, assays must be capable of operation over a range of ambient temperatures. Figure 6 shows operation of the optimized assay from 25 to 40 °C. It can be seen that from 25 to 35 °C, the distance decreased and increased at 40 °C, demonstrating that the impact of thrombin activity dominated over other effects, such as changes in viscosity. Variations in assay response to temperature can be addressed without temperature control using multiple, temperature-dependent assay ranges.

The current assay has been developed and optimized in plasma due to the absence of clinical sample availability during COVID, which currently represents a limitation of demonstration of the device in whole blood. However, previous devices based on polymer microfluidic chips which were optimized in plasma were also found to be suitable for operation in whole blood, and there are no obvious technical limitations for the future demonstration of the current device in a similar manner.^4^

## Conclusions

A paper-based device for measuring fibrinogen in blood plasma has been developed using wax-printed chromatographic paper strips modified with an immobilized thrombin reagent. Clotting of the blood plasma sample was induced by the sample coming into contact with the thrombin reagent and the distance travelled by the sample, and the sample fibrinogen content had an inverse correlative relationship. The device was capable of measuring plasma fibrinogen concentration in the range of 0.5–7 mg/mL in less than 5 min. When packaged in aluminum bags with a desiccant and stored in a fridge or freezer, the device could remain active and functional for 3 weeks. The device was simple, low cost, robust, and sustainable for use in low-resource environments and emergency settings.
